# Protective Effect of the Ethyl Acetate Fraction of *Sargassum muticum* Against Ultraviolet B–Irradiated Damage in Human Keratinocytes

**DOI:** 10.3390/ijms12118146

**Published:** 2011-11-18

**Authors:** Mei Jing Piao, Weon Jong Yoon, Hee Kyoung Kang, Eun Sook Yoo, Young Sang Koh, Dong Sam Kim, Nam Ho Lee, Jin Won Hyun

**Affiliations:** 1 School of Medicine, Jeju National University, Jeju 690-756, South Korea; E-Mails: mjpiao@hanmail.net (M.J.P.); pharmkhk@jejunu.ac.kr (H.K.K.); eunsyoo@jejunu.ac.kr (E.S.Y.); yskoh7@jejunu.ac.kr (Y.S.K.); 2 Jeju Biodiversity Research Institute, Jeju Technopark, Jeju 699-943, South Korea; E-Mails: yyjkl@jejuhidi.or.kr (W.J.Y.); ecklonia@jejutp.or.kr (D.S.K.); 3 Department of Chemistry, College of Natural Sciences, Jeju National University, Jeju 690-756, South Korea; E-Mail: namho@jejunu.ac.kr

**Keywords:** *Sargassum muticum*, ultraviolet B, reactive oxygen species, HaCaT cells, apoptosis

## Abstract

The aim of this study was to investigate the cytoprotective properties of the ethyl acetate fraction of *Sargassum muticum* (SME) against ultraviolet B (UVB)-induced cell damage in human keratinocytes (HaCaT cells). SME exhibited scavenging activity toward the 1,1-diphenyl-2-picrylhydrazyl radicals and hydrogen peroxide (H_2_O_2_) and UVB-induced intracellular reactive oxygen species (ROS). SME also scavenged the hydroxyl radicals generated by the Fenton reaction (FeSO_4_ + H_2_O_2_), which was detected using electron spin resonance spectrometry. In addition, SME decreased the level of lipid peroxidation that was increased by UVB radiation, and restored the level of protein expression and the activities of antioxidant enzymes that were decreased by UVB radiation. Furthermore, SME reduced UVB-induced apoptosis as shown by decreased DNA fragmentation and numbers of apoptotic bodies. These results suggest that SME protects human keratinocytes against UVB-induced oxidative stress by enhancing antioxidant activity in cells, thereby inhibiting apoptosis.

## 1. Introduction

Ultraviolet (UV) radiation is the primary environmental cause of skin damage, leading to conditions such as skin carcinogenesis, inflammation, solar erythema and premature senescence [1–7]. Many harmful effects of short-wavelength UVB ray (290–320 nm) are associated with the production of reactive oxygen species (ROS) (e.g., singlet oxygen, superoxide anions, hydroxyl radicals and hydrogen peroxide) [8,9]. An enzymatic antioxidant defense system composed of catalase (CAT) and superoxide dismutase (SOD) is therefore crucial for the protection of the skin from UVB-induced oxidative stress [10–12]. Severe depletion of endogenous skin antioxidants during oxidative stress following prolonged exposure to UVB radiation results in insufficient sun protection, cellular damage and eventual apoptotic cell death [13]. Thus, the current approach to safeguarding human skin against UVB-induced oxidative damage relies on the avoidance of excessive exposure to sunlight and the use of sun-blocks. However, topical and oral supplementation of natural compounds or products may also complement these strategies [14–16].

A brown algae, *Sargassum muticum*, is widely distributed on the seashores of the southern and eastern parts of Korea. The extracts of *S. muticum* demonstrated various biological activities, including antioxidant, antimicrobial and anti-inflammatory properties [17,18]. However, little is known about the protective effects of the *S. muticum* against UVB radiation. The present study therefore examined the ability of extracts of *S. muticum* to shield human HaCaT keratinocytes from UVB-induced oxidative stress.

## 2. Results

### 2.1. Scavenging Effect of SME toward Free Radicals

**Figure 1:**
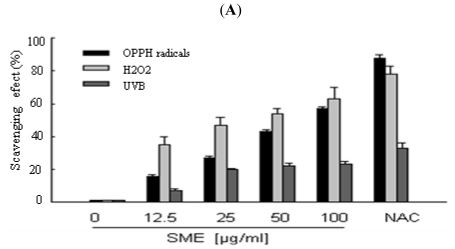

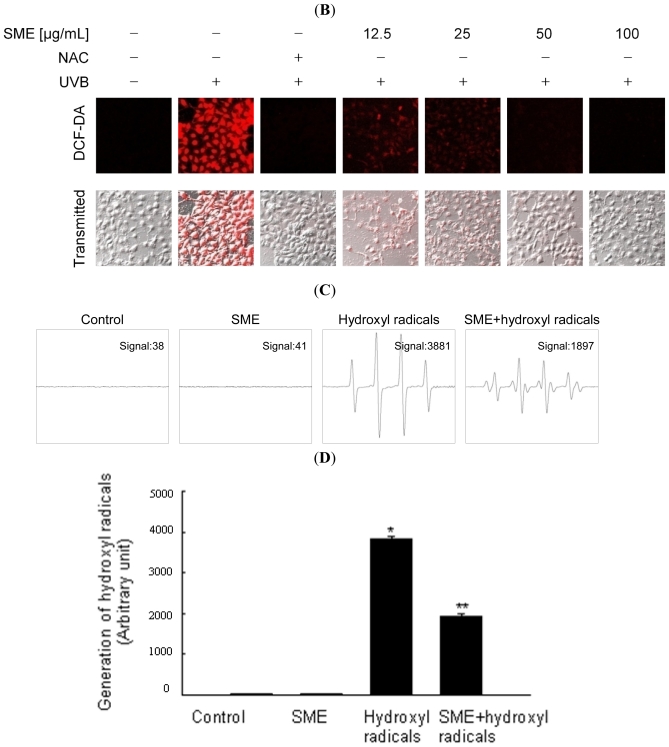

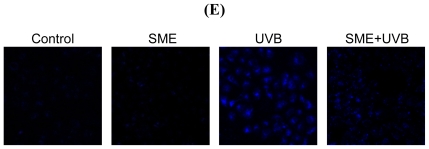
Scavenging effects of SME toward radicals. (**A**) The level of the DPPH radicals was measured spectrophotometrically at 520 nm. Intracellular ROS levels generated by H_2_O_2_ and UVB were detected using a spectrofluorometer after 2′,7′-dichlorodihydrofluorescein diacetate (DCF-DA) staining; (**B**) Representative confocal images illustrate the increase in the red fluorescence intensity of the ROS-induced DCF in UVB-exposed cells compared with control (non-exposed) cells. Hydroxyl radicals generated by the Fenton reaction (H_2_O_2_ + FeSO_4_) were reacted with 5, 5-dimethyl-1-pyrroline-N-oxide (DMPO), and the resultant DMPO/·OH adducts were detected by ESR spectrometry. Results are shown as representative peak data in (**C**) and as a histogram in (**D**); * indicates significantly different from control (*p* < 0.05) and ** indicates significantly different from the hydroxyl radical group (*p* < 0.05); (**E**) Representative confocal images illustrate the increase in the blue fluorescence intensity of the DPPP oxide produced by lipid hydroperoxides in UVB-exposed cells compared with control cells. (**A**)

S. *muticum* was extracted with 80% ethanol. The extract was then successively partitioned to yield *n*-hexane, dichloromethane, ethyl acetate, butanol and water fractions. The ROS scavenging effect of the ethyl acetate fraction (SME) was more effective than the other fractions (data not shown). SME scavenged the 1,1-diphenyl-2-picrylhydrazyl (DPPH) radicals in a concentration-dependent manner; the amount of DPPH radicals scavenged was 16% at 12.5 μg/mL SME, 27% at 25 μg/mL, 43% at 50 μg/mL, and 57% at 100 μg/mL. These results may be compared with 88% scavenging effect for the positive control, N-acetyl cysteine (NAC, 2 mM) (Figure 1A black bars). The H_2_O_2_-induced intracellular ROS scavenging activity of SME was also evaluated and found to be 35% at 12.5 μg/mL, 47% at 25 μg/mL, 54% at 50 μg/mL, and 63% at 100 μg/mL. The corresponding scavenging activity for NAC was 78% (Figure 1A light gray bars). Finally, the UVB-induced intracellular ROS scavenging activity of SME was investigated and found to be 7% at 12.5 μg/mL, 20% at 25 μg/mL, 22% at 50 μg/mL, and 23% at 100 μg/mL, whereas NAC scavenged 33% of the UVB-induced ROS (Figure 1A dark gray bars). SME did not show any cytotoxicity against human HaCaT keratinocytes at 12.5, 25, 50, and 100 μg/mL (data not shown). Based on the results from (Figure 1A), 100 μg/mL was chosen as the optimal dose of SME for further investigation. Fluorescence microscopy analysis of the red fluorescence intensity corresponding to the 2′,7′-dichlorofluorescein (DCF) produced by ROS showed that SME decreased the ROS signal upon UVB radiation, thus reflecting a reduction in ROS generation (Figure 1B). The scavenging effect of SME toward the hydroxyl radical was next measured by electron spin resonance (ESR) spectrometry. The ESR data showed that the hydroxyl radicals signal increased up to a value of 3881 in the Fenton reaction (H_2_O_2_ + FeSO_4_) system; however, SME decreased the hydroxyl radical signal to a value of 1897 (Figures 1C and D). Lipid peroxidation was monitored using diphenyl-1-pyrenylphosphine (DPPP). DPPP reacts with lipid hydroperoxides to produce the highly fluorescent product, DPPP oxide [19]. The fluorescence intensity of DPPP oxide was enhanced in UVB-irradiated cells and greatly reduced in cells treated with SME (Figure 1E).

### 2.2. Effect of SME on Antioxidant Enzymes

**Figure 2:**
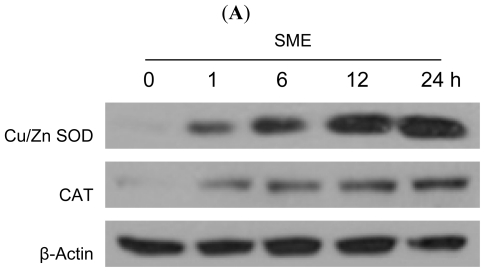

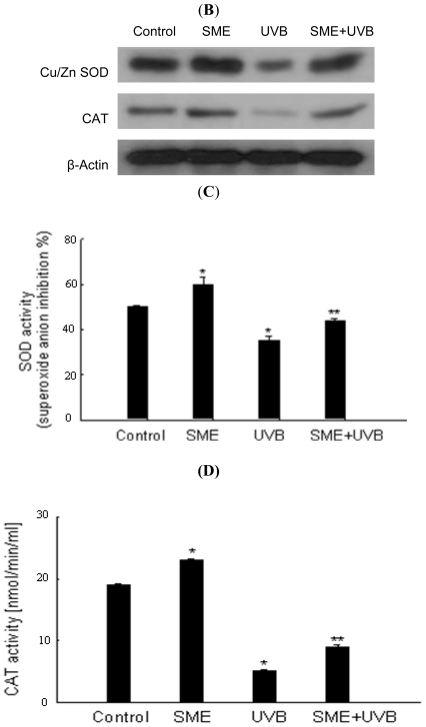
The effects of SME on the protein expression level and activity of antioxidant enzymes. (**A and B**) Cell lysates were electrophoresed in SDS-PAGE gels and transferred to nitrocellulose membrane. Cu/Zn SOD and CAT were detected on immunoblots via reaction with their specific antibodies; (**C**) SOD activity was measured using a colorimetric assay kit, and activity was represented as the percent inhibition of the superoxide anions. * indicates significantly different from control (*p* < 0.05), and ** indicates significantly different from UVB-exposed cells (*p* < 0.05); (**D**) CAT activity was measured using a colorimetric assay kit, and activity was represented as nmol/min/mL. * indicates significantly different from control (*p* < 0.05), and ** indicates significantly different from UVB-exposed cells (*p* < 0.05).

To investigate whether the radical scavenging activity of SME was mediated by antioxidant enzymes, the protein expression level of Cu/Zn SOD and CAT in SME-treated cells were measured. As shown in Figure 2A, SME increased the protein expression levels of both Cu/Zn SOD and CAT in a time-dependent manner compared with the expression level in control cells. In UVB-exposed cells, the protein expression of Cu/Zn SOD and CAT was decreased (Figure 2B). However, SME treatment restored enzyme expression in these cells (Figure 2B). The activity of SOD and CAT in UVB-irradiated cells was also reduced compared with activity in the control cells, but SME restored enzyme activity (Figures 2C and D).

### 2.3. Effect of SME against Cell Death Induced by UVB Radiation

**Figure 3:**
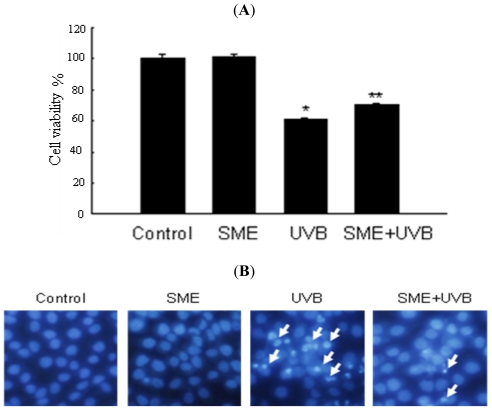

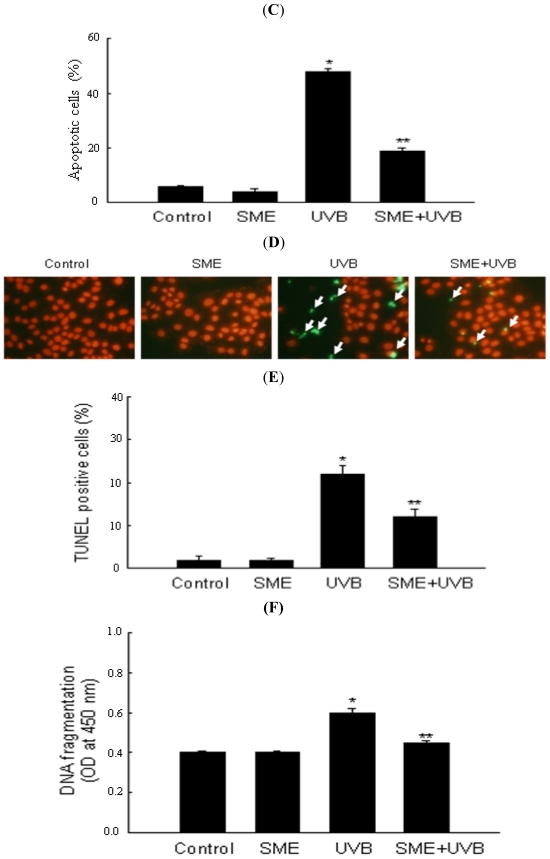
The effect of SME on UVB-induced cell death. Cells were treated with SME at a concentration of 100 μg/mL, exposed to UVB radiation 1 h later and incubated for 48 h. (**A**) Cell viability was determined by the MTT assay. * indicates significantly different from control (*p* < 0.05), and ** indicates significantly different from UVB-exposed cells (*p* < 0.05); **(B**) Apoptotic bodies (arrows) were observed under a fluorescence microscope in cells stained with Hoechst 33342 and (**C**) quantified. * indicates significantly different from control (*p* < 0.05), and ** indicates significantly different from UVB-exposed cells (*p* < 0.05); (**D**) Apoptotic cells were detected by the TUNEL staining assay and (**E**) quantified. The arrows point to TUNEL-positive cells; (**F**) DNA fragmentation was quantified using a cytoplasmic histone-associated DNA fragmentation kit. * indicates significantly different from control (*p* < 0.05), and ** indicates significantly different from UVB-exposed cells (*p* < 0.05).

To investigate whether SME has cytoprotective activity in UVB-irradiated cells, the viability of cells exposed to UVB was first measured using the MTT assay. Indeed, cell viability was significantly increased from 61% in UVB (150 mJ/cm^2^)-irradiated cells to 70% in UVB-irradiated cells treated with SME (Figure 3A). Next, direct association of apoptosis with UVB-induced cell death was investigated, as well as inhibition of UVB-induced cell death by SME. Apoptotic bodies, TUNEL-positive cells and DNA fragmentation, which are all indicators of apoptosis, were observed in UVB-irradiated cells. Intact nuclei were observed in control cells, whereas significant nuclear fragmentation was observed in UVB-exposed cells. However, nuclear fragmentation was dramatically reduced in UVB-irradiated cells that were treated with SME (Figures 3B and C). Moreover, while the levels of TUNEL-positive cells and cytoplasmic histone-associated DNA fragmentation were higher in UVB-irradiated cells compared with control cells, the levels of TUNEL-positive cells and DNA fragmentation were significantly decreased in UVB-irradiated cells that were treated with SME (Figures 3D, E and F).

## 3. Discussion

UVB-induced ROS have hazardous effects on the skin, including sunburn, photoaging and skin cancer. UVB rays interact with cellular chromophores and photosensitizers, resulting in the generation of singlet oxygen, superoxide anions, hydroxyl radicals and hydrogen peroxide [20]. The data presented herein clearly demonstrate that UVB radiation triggered an increase in the intracellular ROS level in HaCaT cells. In this study, SME treatment significantly reduced ROS generation in UVB-irradiated cells, apparently due to the ROS scavenging property of the extract. The data indicate that SME scavenged intracellular ROS, as well as DPPH radicals and hydroxyl radicals. SME also inhibited lipid peroxidation. However, SME did not scavenge superoxide anions generated by the xanthine/xanthine oxidase system, which were detected using ESR (data not shown). Kim *et al.* reported that SME contained polyphenol (347 mg/g), a well-known antioxidant compound, as assessed the Folin-Denis method [17]. García-Casal *et al.* reported that *Sargassum* species contained a high polyphenol content in comparison with *Ulva* species and *Porphyra* species. *Sargassum* species also showed a higher antioxidant capacity than did the other two species [21]. Therefore, the high polyphenol content in SME could be partly responsible for its antioxidant effect. On the other hand, brown algae reportedly contain phlorotannins. Phlorotannins are marine algal polyphenols and polymers of phloroglucinol [22] that are suggested to exhibit antioxidant effect against ROS [23–25].

Previous reports show that enhanced oxidative stress induced by UVB radiation is accompanied by decreases in the activity of the antioxidant enzymes SOD and CAT [10]. SME treatment increased both the level of Cu/Zn SOD and CAT protein expression and the activity of SOD and CAT in UVB-irradiated cells. Thus, the enzyme-enhancing actions of SME may also contribute to its beneficial effects against cell damage caused by UVB exposure. The oxidative stress induced by UVB radiation results in injury of skin cells and, ultimately, apoptosis [26]. SME treatment suppressed UVB radiation-induced cell death via inhibition of apoptosis, as shown by decreased DNA fragmentation and numbers of apoptotic bodies. Recently, Bozzo *et al*. reported that at the same UVB doses primary human keratinocytes induced a higher percentage of apoptosis than in immortalized HaCaT keratinocyte in an *in vitro* system. Therefore, further studies are required to investigate the cytoprotective effect of SME against UVB radiation in primary human keratinocytes [27]. In conclusion, the results of the current study suggest that the cytoprotective effect of SME against UVB-induced oxidative damage in HaCaT keratinocytes may involve two mechanisms of action: (1) a direct scavenging effect on free radicals such as DPPH radicals and hydroxyl radicals and (2) an indirect effect via the induction of antioxidant enzymes activities such as SOD and CAT.

## 4. Experimental Section

### 4.1. Solvent Extraction of Sargassum Muticum

*Sargassum muticum* specimens were collected on Jeju Island (South Korea). Voucher specimen number JBR-253 was deposited at the herbarium of the Jeju Biodiversity Research Institute (Jeju, South Korea). The dried material (62 g) was extracted with 80% ethanol at room temperature for 24 h and was then evaporated under a vacuum. The evaporated 80% ethanol extract (12.1 g, 19.5%) was suspended in water and partitioned with four solvents: *n*-hexane, dichloromethane, ethyl acetate and butanol; this partitioning was repeated three times. These four solvent partitions yielded *n*-hexane (1.7 g, 13.1%), dichloromethane (0.7 g, 5.4%), ethyl acetate (0.1 g, 0.8%), butanol (1.2 g, 9.2%) and water (8.2 g, 63.1%) fractions.

### 4.2. Reagents

1,1-Diphenyl-2-picrylhydrazyl (DPPH) radical, N-acetyl cysteine (NAC), 5,5-dimethyl-1-pyrroline- N-oxide (DMPO), 2′,7′-dichlorodihydrofluorescein diacetate (DCF-DA), [3-(4,5-dimethylthiazol-2-yl)- 2,5-diphenyltetrazolium] bromide (MTT) and Hoechst 33342 dye were purchased from Sigma Chemical Company (St. Louis, MO, USA). Cu/Zn SOD and CAT antibodies were purchased from Biodesign International (Saco, ME, USA). Diphenyl-1-pyrenylphosphine (DPPP) was purchased from Molecular Probes (Eugene, OR, USA). All other chemicals and reagents were of analytical grade.

### 4.3. Cell Culture

Human keratinocytes (HaCaT cells) were obtained from the Amore Pacific Company (Gyeonggi-do, South Korea). Cells were maintained at 37 °C in an incubator with a humidified atmosphere of 5% CO_2_. Cells were cultured in Dulbecco’s modified Eagle’s medium containing 10% heat-inactivated fetal calf serum, streptomycin (100 μg/mL) and penicillin (100 units/mL).

### 4.4. DPPH Radical Scavenging Activity

The ethyl acetate fraction of *S. muticum* (SME) at concentrations of 12.5, 25, 50 and 100 μg/mL were added to a 1 × 10^−4^ M solution of DPPH in methanol. The resulting reaction mixture was shaken vigorously. After 3 h, the amount of DPPH remaining was measured at 520 nm using a spectrophotometer.

### 4.5. Intracellular ROS Scavenging Activity

The DCF-DA method was used to detect intracellular ROS levels in HaCaT keratinocytes [28]. For detection of ROS in H_2_O_2_-treated cells, cells were seeded at a density of 1.5 × 10^5^ cells/well. Sixteen hours after plating, cells were treated with SME at concentrations of 12.5, 25, 50 and 100 μg/mL. After 30 min, H_2_O_2_ (1 mM) was added to the plate. Cells were incubated for an additional 30 min at 37 °C. For detection of ROS in UVB-exposed cells, cells were treated with SME as described above. After one hour, cells were exposed to UVB radiation at a dose of 150 mJ/cm^2^. The UVB source was a CL-1000M UV Crosslinker (UVP, Upland, CA, USA), which was used to deliver an energy spectrum of UVB radiation (280–320 nm; peak intensity 302 nm). Cells were incubated for an additional 24 h at 37 °C. After the addition of DCF-DA solution (25 μM) for 10 min, the fluorescence of 2′,7′-dichlorofluorescein was detected using a PerkinElmer LS-5B spectrofluorometer (PerkinElmer, Waltham, MA, USA). Image analysis for the generation of intracellular ROS was achieved by seeding the cells on a cover-slip loaded six-well plate at 2 × 10^5^ cells/well. Sixteen hours after plating, the cells were treated with SME at a concentration of 100 μg/mL. One hour following SME treatment, the plate was irradiated with UVB. Twenty four hours later, DCF-DA (100 μM) was added to each well, and cells were incubated for an additional 30 min at 37 °C. After washing with phosphate-buffered saline (PBS), the stained cells were mounted onto a microscope slide in mounting medium (DAKO, Carpinteria, CA, USA). Images of the cells were collected using the laser scanning microscope 5 PASCAL program (Carl Zeiss, Jena, Germany) on a confocal microscope.

### 4.6. Detection of Hydroxyl Radicals

Hydroxyl radicals were generated by the Fenton reaction (H_2_O_2_ + FeSO_4_) and then reacted with a nitrone spin trap, DMPO. The resultant DMPO/·OH adducts was detected using a JES-FA electron spin resonance (ESR) spectrometer (JEOL, Tokyo, Japan) [29,30]. The ESR spectrum was recorded at 2.5 min after mixing with a phosphate buffer solution (pH 7.4) containing 0.2 mL each of 0.3 M DMPO, 10 mM FeSO_4_, 10 mM H_2_O_2_, and SME (100 μg/mL). The ESR spectrometer parameters were set at a magnetic field of 336 mT, power of 1.00 mW, frequency of 9.4380 GHz, modulation amplitude of 0.2 mT, gain of 200, scan time of 0.5 min, scan width of 10 mT, time constant of 0.03 sec and a temperature of 25 °C.

### 4.7. Lipid Peroxidation Assay

Lipid peroxidation was estimated using DPPP, a fluorescent probe [19]. After the cells were incubated with 5 μM DPPP for 15 min in the dark, they were treated with SME. The DPPP fluorescence images were analyzed using a Zeiss Axiovert 200 inverted microscope at an excitation wavelength of 351 nm and an emission wavelength of 380 nm.

### 4.8. Western Blot Analysis

Cells were treated with SME (100 μg/mL) for 1, 6, 12 and 24 h; alternatively, cells were treated with SME (100 μg/mL) for 1 h and then exposed to UVB and incubated at 37 °C for 24 h. The harvested cells were then lysed on ice for 30 min in lysis buffer (100 μL) containing 120 mM NaCl, 40 mM Tris (pH 8.0) and 0.1% NP 40. Cells were centrifuged at 13,000 × *g* for 15 min. The supernatants were then collected from the lysates, and the protein concentrations were determined. Aliquots of the lysates (40 μg of protein) were boiled for 5 min and electrophoresed on a 10% SDS-polyacrylamide gel. The electrophoresed proteins were transferred onto nitrocellulose membranes, and the membranes were subsequently incubated with primary antibodies against Cu/Zn SOD and CAT. The membranes were further incubated with secondary immunoglobulin-G-horseradish peroxidase conjugates (Pierce, Rockford, IL, USA). The protein bands were detected using an enhanced chemiluminescence Western blotting detection kit (Amersham, Little Chalfont, Buckinghamshire, UK).

### 4.9. SOD Activity

Cells were treated with SME at a concentration of 100 μg/mL for 24 h. One hour later, they were exposed to UVB and incubated at 37 °C for 24 h. SOD activity was measured using a colorimetric assay kit (Abcam, Cambridge, MA, USA) according to the manufacturer’s protocol. The kit utilized the tetrazolium salt WST-1, which produces a water-soluble formazan dye upon reduction with the superoxide anions. The formazan dye product was detected at 450 nm. SOD activity was calculated on the basis of the percent inhibition of the superoxide anions.

### 4.10. CAT Activity

CAT activity was measured using a colorimetric assay kit (Cayman Chemical Co., Ann Arbor, MI, USA) according to the manufacturer’s protocol. Briefly, CAT reacts with methanol, an electron donor, in the presence of H_2_O_2_ to produce formaldehyde. Formaldehyde is then measured colorimetrically at 540 nm using 4-amino-3-hydrazino-5-mercapto-1,2,4-triazole as the chromogen. CAT activity was expressed in nmol/min/mL.

### 4.11. Cell Viability Assay

Cells were seeded in a 96-well plate at a concentration of 1 × 10^5^ cells/mL. Sixteen hours after plating, SME was added at a concentration of 100 μg/mL and cells were exposed to UVB radiation at a dose of 150 mJ/cm^2^ one hour later and incubated at 37 °C for 48 h. Fifty microliter of MTT stock solution (2 mg/mL) was added to each well to yield a total reaction volume of 200 μL. After incubating the cells for 4 h, the plate was centrifuged at 800 × *g* for 5 min, and the supernatants were aspirated. The formazan crystals in each well were dissolved in dimethylsulfoxide (150 μL), and the absorbance at 540 nm was read on a scanning multi-well spectrophotometer [31].

### 4.12. Nuclear Staining with Hoechst 33342

Cells were treated with SME at a concentration of 100 μg/mL and exposed to UVB one hour later. Cells were incubated for an additional 48 h at 37 °C. The DNA-specific fluorescent dye Hoechst 33342 (1.5 μL of a 10 mg/mL stock solution) was added to each well, and the cells were incubated for 10 min at 37 °C. The stained cells were then visualized under a fluorescence microscope equipped with a CoolSNAP-Pro color digital camera to examine the degree of nuclear condensation, and the apoptotic cells were quantified.

### 4.13. Terminal Deoxynucleotidyl Transferase-Mediated Digoxigenin-dUTP Nick end Labeling (TUNEL) Assay

The TUNEL assay was performed using an *in situ* cell death detection kit (Roche Diagnostics, Mannheim, Germany) according to the manufacturer’s instructions [32]. Briefly, image analysis for the TUNEL assay was achieved by seeding cells on chamber slides at a density of 2 × 10^5^ cells/well. Sixteen hours after plating, cells were treated with SME. Forty eight hours later, chamber slides were fixed with 4% paraformaldehyde for 1 h at 15–25 °C, and cells were permeabilized for 2 min in 0.1% sodium citrate solution containing 0.1% Triton X-100. After washing in PBS, sections were incubated with the TUNEL reaction mixture for 1 h at 37 °C. After washing with PBS, the stained cells were mounted onto a microscope slide in mounting medium (DAKO, Carpinteria, CA, USA). Cells were then observed under a fluorescent microscope (Olympus IX 70, Olympus Optical Co., Tokyo, Japan) and quantified.

### 4.14. DNA Fragmentation

Cellular DNA fragmentation in HaCaT cells was assessed by analyzing cytoplasmic histone-associated DNA fragmentation using a kit from Roche Diagnostics (Portland, OR, USA) according to the manufacturer’s instructions.

### 4.15. Statistical Analysis

All values were expressed as means ± standard error. The results were subjected to an analysis of variance (ANOVA) using the Tukey’s test to analyze difference. A *p*-value of <0.05 was considered statistically significant.

## 5. Conclusion

SME protected cells against UVB radiation via free radical scavenging capacity, induction of antioxidant enzymes and inhibition of apoptosis. Therefore, these results suggest that SME may be developed as a protective agent against UVB damage.
